# Environmental microbial community stabilization and plant growth enhancement by combined carbon nanomaterials and nitrification inhibitor in soil contaminated with polyvinyl chloride

**DOI:** 10.1128/aem.01568-25

**Published:** 2025-12-04

**Authors:** Manyun Zhang, Efrizal Efrizal, Islah Hayati, Xinhong Gan, Benliang Zhao, Tao Guo, Minzhe Zhou, Wenhui Tang, Bin Ma

**Affiliations:** 1 College of Environmental Science and Engineering, Yangzhou University 38043, Yangzhou, Jiangsu, China; 2 Centre for Planetary Health and Food Security, School of Environment and Science, Griffith University 5723, Brisbane, Queensland, Australia; 3 College of Environment and Ecology, Hunan Agricultural University 12575, Changsha, China; 4 Faculty of Agriculture, Jambi University 175446, Jambi-Sumatera, Indonesia; 5 Institute of Biological Sciences, Faculty of Science, Universiti Malaya 37447, Kuala Lumpur, Malaysia; 6 Department of Agro Eco-Technology Faculty of Agriculture, Jambi University 175446, Jambi, Indonesia; 7 State Environmental Protection Key Laboratory of Soil Environmental Management and Pollution Control, Nanjing Institute of Environmental Science, Ministry of Ecology and Environment (MEE) of China, Nanjing, China; 8 Key Laboratory of Agro-environments in Tropics, Ministry of Agriculture and Rural Affairs/Guangdong Engineering Research Center for Modern Eco-agriculture and Circular Agriculture, South China Agricultural University 12526, Guangzhou, China; 9 Sichuan Agricultural University, College of Resources 12529, Chengdu, China; University of Georgia Center for Food Safety, Griffin, Georgia, USA

**Keywords:** PVC, MWCNTs, nanodiamonds, DMPP, biomass, endophytic bacterial and fungal communities

## Abstract

Microplastics are widely distributed in soil ecosystems, posing significant threats to soil health and plant growth. This study investigated the role of environmental microbial communities in mitigating the toxic effects of microplastics on soil-plant systems. Carbon nanomaterials and nitrification inhibitor 3,4-dimethylpyrazole phosphate (DMPP) can regulate soil microbial communities and nitrogen transformation enzyme activities. Microplastics polyvinyl chloride (PVC) significantly reduced the plant biomass and soluble sugar by 32.4% and 39.2%, respectively. However, combined multi-walled carbon nanotubes (MWCNTs), nanodiamonds, and DMPP alleviated those toxic effects. PVC significantly declined the soil bacterial community stabilization, and extra nanomaterials recovered soil bacterial community stabilization to blank control level. Moreover, extra nanomaterials exhibited double-edged sword effects on the endophytic microbes, which significantly inhibited the *α*-diversities and stabilizations of the endophytic bacterial community but promoted the *α*-diversities and stabilizations of the endophytic fungal community. Plant biomass was significantly correlated with the soil pH, plant soluble sugar and nitrate content, and *α*-diversities and stabilizations of the endophytic bacterial and fungal communities. These findings highlight the pivotal role of environmental microbial communities in mediating the response of plants to microplastic contamination and offer valuable insights for alleviating the toxic effects of microplastic contamination.

**IMPORTANCE:**

Carbon nanomaterials and nitrification inhibitor 3,4-dimethylpyrazole phosphate (DMPP) can serve as plant growth regulators and regulate soil health. Combined multi-walled carbon nanotubes, nanodiamonds, and DMPP could relieve the toxic impacts of polyvinyl chloride (PVC) on soil bacterial community stabilization, plant biomass, and quality. The extra nanomaterials exhibited double-edged sword effects in that they significantly inhibited the endophytic bacterial community *α*-diversities and stabilizations but promoted the endophytic fungal community *α*-diversities and stabilizations. This study would provide theoretical and practical foundations to relieve the toxic impacts of microplastics on the soil-plant system.

## INTRODUCTION

Microplastics are widely distributed in soil ecosystems and pose significant threats to soil health and plant growth (1). The microplastics can alter soil physical properties and biochemical processes (soil microbial community and enzyme activities) (2, 3). The microplastics can also inhibit plant growth and generate ecotoxicity and genotoxicity (4). For instance, the microplastics influence plant growth by modulating leaf physiological processes (5), and the microplastic phenanthrene significantly reduces the chlorophyll content in the leaf, inhibiting plant growth (6). Microplastics-induced variations in soil productivity can also indirectly regulate plant growth (7). These multifaceted impacts present substantial challenges to sustainable agriculture and the preservation of ecosystem functions. Prior research focused on the effects of microplastics on soil microbial communities and rhizosphere processes (8, 9), but the investigations into their direct influence on endophytic microbial communities remain limited. Given the crucial role of endophytic communities in plant development, we hypothesized that microplastics may negatively affect plant growth by modulating endophytic microbial communities and plant physiological indicators.

Soil amendments and growth indicators potentially alleviated the negative impacts of microplastics on the soil-plant system (10). Nanomaterials, characterized by their exceptional specific surface area, strong adsorption capacity, and antioxidant properties, have demonstrated significant potential in pollutant remediation (11, 12). Nanomaterials could enhance soil phytochemistry properties, optimize nutrient uptake, and modulate microbial community to promote plant growth (13). Carbon nanomaterials can serve as plant growth regulators, beneficially influencing plant growth (14). For instance, the carbon nanotubes can penetrate seed coats, facilitate water absorption, and influence seed germination and growth (15). Additionally, carbon nanotubes can also capture or degrade pollutants, thereby mitigating their inhibitory effects on key microorganisms, and they can also regulate soil microbial communities and nitrogen transformation enzyme activities, accelerating nitrogen mineralization and nitrification processes (16). However, nanomaterials could exert dislocating effects on plants and subsequent food chains. For example, rare earth oxide nanomaterials could release Ce^3+^, which can form insoluble CePO_4_ precipitates with phosphate, thereby potentially immobilizing soil phosphorus and impairing plant phosphorus uptake (17). Moreover, gold nanoparticles can be absorbed by tobacco and transferred to herbivorous Manduca sexta larvae, with concentrations in the larvae significantly exceeding those in tobacco, indicating trophic magnification (18). This discovery warns that as the number of products containing nanomaterials increases, humans and ecological receptors may face risks of long-term exposure (18). Hence, it is essential to probe the specific mechanisms of nanomaterials' impacts on soil-plant systems. Thus, we proposed that the carbon nanomaterials can relieve the toxic impacts of microplastics on soil-plant systems through improving nitrogen availability, optimizing soil microbial communities, and facilitating nutrient cycling processes.

As for the main nitrogen management measure, nitrification inhibitors, such as 3,4-dimethylpyrazole phosphate (DMPP), can regulate soil nitrogen transformation, suppress nitrification, and enhance nutrient utilization efficiency in plants (19). Our previous study found that the DMPP and polyvinyl chloride had antagonist impacts on nitrous oxide emission rates and soil *Firmicutes* (20). Therefore, we proposed that nitrification inhibitors can also relieve the toxic impacts of microplastics on soil-plant systems by influencing nitrogen cycling processes and regulating microbial communities. Despite previous studies emphasizing the contributions of nitrification inhibitors and carbon nanomaterials to soil-plant systems, research on their combined effects remains limited. In this study, we innovatively chose the multi-walled carbon nanotubes (MWCNTs), nanodiamonds, and DMPP to handle microplastic-induced disruptions, with two contributions: (i) nanomaterials can adsorb/neutralize microplastics and their byproducts, while DMPP alleviates microplastic-induced nitrogen loss (19, 21, 22), creating a “pollutant neutralization-nutrient retention” dual mechanism; (ii) nanomaterials could modulate soil and endophytic microbial communities, enhancing soil nutrient cycling and promoting plant health, while DMPP could improve nitrogen availability to strengthen plant physiological resilience (13, 23). Hence, we hypothesized that the combined MWCNTs/nanodiamonds and DMPP could alleviate microplastic-induced toxicity by (i) enhancing soil nitrogen retention via DMPP-mediated nitrification inhibition, (ii) improving endophytic microbial community stability through carbon nanomaterial-driven microbial community modulation, and (iii) reducing plant oxidative stress via the antioxidant properties of nanomaterials.

Due to its small and relatively homogeneous particle diameter and extensive use, polyvinyl chloride was selected as the experimental microplastic. This study examined the combined effects of MWCNTs, nanodiamonds, and DMPP to address microplastics-induced disruptions in soil-plant systems. This study focuses on (i) investigating how microplastics influence plant growth and physiological indicators by altering endophytic microbial communities; (ii) elucidating the effects of carbon nanomaterials in relieving the negative impacts of microplastics on the soil-plant systems; (iii) evaluating the contributions of DMPP on improving soil nitrogen cycling and reducing the adverse impacts of microplastics on plant growth; (iv) exploring the synergistic effects of combining carbon nanomaterials and DMPP in regulating the impacts of microplastics. This study would provide theoretical and practical foundations to relieve the toxic impacts of microplastics on the soil-plant system.

## MATERIALS AND METHODS

### Materials and experimental soil

The basic information on the nitrification inhibitor DMPP and microplastics polyvinyl chloride (PVC) was reported in our previous study (20). The MWCNTs were purchased from Shenzhen SUIHENG Technology Co., Ltd. (Shenzhen, China), with purity ≥95%. Additional characteristics are average diameter (≤7 µm), specific surface area (≥250 m²/g), and bulk density (0.08 g/cm³). Nanodiamonds were purchased from Shenzhen SUIHENG Technology Co., Ltd. (Shenzhen, China), with a particle size = 30 nm and a purity of 95%. The experimental soil was sampled from the Changan research base of Hunan Agricultural University (N28°10′, E113°4′). After removing litter residues and debris, the test soil was air-dried, ground, and passed through a 2 mm sieve before the experiment. The soil background values of this experiment soil are: soil pH (5.34), NH_4_^+^–N (36.76 mg kg^−1^), NO_3_^−^–N (73.92 mg kg^−1^), and available phosphorus (26.97 mg kg^−1^).

### Experiment design

The experiment comprised seven groups with four replicates: (i) black control (CK); (ii) PVC existence (PVT); (iii) PVC and MWCNTs co-existence (PCNT); (iv) PVC and nanodiamonds co-existence (PCDT); (v) PVC and DMPP co-existence (PDMT); (vi) PVC, MWCNTs, and DMPP co-existence (PCNMT); and (vii) PVC, nanodiamonds, and DMPP co-existence (PCDMT). The PVC rate was 0.5% of soil dry matter, and the DMPP was added at 1.0% of applied urea-N (8, 24). The MWCNTs and nanodiamonds were added at 1.0 mg kg^−1^ dry soil (25).

The plant (cabbage) seeds were surface-sterilized and then germinated in a dark environment (24). Each pot was filled with 2.5 kg of soil with or without PVC inclusion. Fifteen germinating seeds were selected and distributed in each pot. All pots were placed in a room with natural sunlight and ambient temperature. The sowing day was noted as day 0, and then the plants underwent thinning to retain four seedlings per pot (day 14). The fertilizers and DMPP were dissolved in double-distilled water (ddH_2_O) and applied on days 16 and 17, respectively. The urea was applied at 200 mg N kg^−1^ dry soil, and the additional ratio of urea, trisodium phosphate, and potassium chloride fertilizer was 2:1:1 (24). The pots were regularly monitored and watered, with daily light and temperature variations shown in Table S1 at https://doi.org/10.6084/m9.figshare.30681275. The plant and soil samples were collected on day 40 for the following parameter determinations.

### Determinations of soil chemical and enzyme properties

Air-dried soil subsamples were prepared to determine soil pH and available phosphorus (AP), and fresh soil subsamples were prepared to determine soil NH_4_^+^–N and NO_3_^−^–N contents and enzyme activities. Details of the analytic methods of soil abiotic properties are demonstrated in Table S2 at https://doi.org/10.6084/m9.figshare.30681275. Soil *β*-glucosidase, chitinase, acid phosphatase, and arylsulfatase activities were determined by *para*-nitrophenol (*p*NP)-based enzyme assay methods. In brief, the soil samples were incubated with 4 mL of 50 mmol buffer solution and 1 mL of reaction substrates, and the enzymatic reaction was terminated by 0.5 M CaCl_2_ and 1 M NaOH. After centrifuging, the supernatant was collected to determine the absorbance at a wavelength of 410 nm (26, 27). The indophenol blue method was adopted to measure the soil urease activity (27). Briefly, soil samples were sequentially treated with 0.2 mL toluene, 4 mL urea solution, and 8 mL citrate buffer at 37°C for 24 h. The reaction was halted by adding 18 mL of preheated water, and after standing, 0.2 mL supernatant, 3.4 mL ddH_2_O, 0.8 mL sodium phenolate solution, and 0.6 mL sodium hypochlorite solution were successively transferred to centrifuge tube and vortexed. After a 20 min color development period, the mixture was made up to 10.0 mL with ddH_2_O, and the absorbance was determined at a wavelength of 578 nm within 1 h. The assessed approach of soil electron transport system activity (ETSA) was according to the study of Wan et al. (28). The details on substrates and buffer solutions are demonstrated in Table S3 at https://doi.org/10.6084/m9.figshare.30681275.

### Determination of plant biomass, quality, and stress resistance indicators, and ecosystem multifunctionality

The fresh plant samples were cleaned to remove soil and other impurities on their surfaces to count their total fresh weight. The plant samples were subsequently divided into dissimilar sub-parts to analyze plant water contents, quality indicators (soluble protein, nitrate, and soluble sugar contents), stress resistance indicators (proline and malondialdehyde content), and endophytic bacterial and fungal communities. Water content was calculated by drying some fresh subsamples at 105°C for 30 min and subsequently at 65°C until reaching constant weight. More details of the analytic methods of plant quality are demonstrated in Table S4 at https://doi.org/10.6084/m9.figshare.30681275. The ecosystem multifunctionality was calculated by Qiu et al. (29).

### Illumina Miseq sequencing and data analyses

The surface sterilization of fresh plant subsample was performed through the following sequential steps: initial rinsing with the ddH_2_O to dislodge surface-adhered soil and dust particles, followed by successive immersion in 70% ethanol, 2.5% sodium hypochlorite, and a second 70% ethanol treatment, and washing with the ddH_2_O (24). The homogenization protocol was listed as follows: 0.2 g of the plant sample and liquid nitrogen were successively placed into a 2.0 mL centrifuge tube; once the liquid nitrogen had fully evaporated, the plant sample was ground into a powder; subsequently, this powder was added to 1.0 mL of cracking solution and fully blended (30). The soil and endophytic bacterial and fungal DNA were extracted with Fast DNA SPIN Kit, and the extracted DNA was checked by 1.0% agarose gel electrophoresis (24). The amplified processes of bacterial 16S RNA and fungal ITS were described by Zhang et al. (24). The purified PCR products were sequenced on the Illumina MiSeq platform (Majorbio, China) with a coverage of 97% per sample. Details about the raw sequences for each sample are demonstrated in Tables S5 and S6 at https://doi.org/10.6084/m9.figshare.30681275. Alpha diversity analyses were conducted at the operational taxonomic unit (OTU) level. The bacterial and fungal OTUs were compared to the SILVA (http://www.arb-silva.de) and PR2 (http://github.com/vaulot/pr2_database) databases to present the taxonomic compositions of bacterial and fungal communities, respectively. Principal coordinates analysis was used to analyze the changes in bacterial and fungal community compositions in different samples. The screened OTU data (relative abundance >0.1%) were conducted based on the Spearman correlation matrix by the WGCNA package, and the co-occurrence network diagrams were present in Gephi software with the Fruchterman-Reingold layout. The “*igraph*” package was utilized to calculate the topological features of co-occurrence networks.

### Statistical analysis

One-way analysis of variance with the Duncan multiple range test was employed to assess the significant differences among the seven groups at a significance level of *P* <0.05. Pearson’s correlation was performed to indicate the linear correlations among different parameters.

The original data were standardized and subjected to dimensionality reduction for pathway analysis via the maximum likelihood method. Path analysis explored the linkages among plant parameters, soil chemical properties, enzyme activities, soil, and endophytic microbial communities.

## RESULTS

### Soil chemical and enzyme properties and ETSA

Nanomaterial and DMPP amendments counteracted the PVC-induced soil acidification (*P* < 0.05), with pH increases ranging from 0.17 (PCNMT) to 0.35 units (PCNT) relative to PVT controls (5.01 ± 0.03) (Table 1). The PCDMT group reduced NH_4_^+^–N availability by 23.4%, and the PCNT, PCDT, PCNMT, and PCDMT groups significantly lowered PVC-induced NO_3_^−^–N accumulation. The available phosphorus contents in the CK, PVT, PCNT, PCDT, PDMT, PCNMT, and PCDMT were 21.6 ± 1.40, 19.9 ± 3.30, 18.5 ± 0.23, 20.0 ± 2.42, 19.9 ± 1.42, 17.2 ± 1.84, and 21.4 ± 1.41 mg kg^−1^ dry soil, respectively.

**TABLE 1:** The soil abiotic and enzyme properties in the different treatments^*a*^

Abiotic and enzyme properties	Treatments						
	CK	PVT	PCNT	PCDT	PDMT	PCNMT	PCDMT
pH	5.09 ± 0.03 c	5.01 ± 0.03 c	5.36 ± 0.06 a	5.06 ± 0.07 c	5.25 ± 0.05 b	5.18 ± 0.05 b	5.22 ± 0.07 b
Organic matter (g kg^−1^ dry soil)	35.0 ± 3.34 b	37.4 ± 3.01 ab	35.9 ± 1.06 b	41.0 ± 3.11 a	38.7 ± 2.45 ab	34.8 ± 2.02 b	38.6 ± 1.54 ab
NH_4_^+^–N (mg kg^−1^ dry soil)	127 ± 9.30 ab	121 ± 6.73 ab	113 ± 1.76 bc	118 ± 11.1 bc	134 ± 10.2 a	120 ± 2.59 bc	105 ± 14.2 c
NO_3_^−^–N (mg kg^−1^ dry soil)	2.38 ± 0.67 b	9.68 ± 1.52 a	2.48 ± 0.89 b	1.70 ± 0.75 bc	0.93 ± 0.13 c	0.73 ± 0.22 c	1.71 ± 0.21 bc
Available P (mg kg^−1^ dry soil)	21.6 ± 1.40 a	19.9 ± 3.30 ab	18.5 ± 0.23 ab	20.0 ± 2.42 ab	19.9 ± 1.42 ab	17.2 ± 1.84 b	21.4 ± 1.41 a
*β*-glucosidase activity (mg *p*NP kg^−1^ dry soil d^−1^)	556 ± 18.9 a	511 ± 35.7 a	523 ± 22.7 a	551 ± 70.7 a	536 ± 19.7 a	523 ± 27.9 a	529 ± 18.1 a
Chitinase activity (mg *p*NP kg^−1^ dry soil d^−1^)	477 ± 19.3 ab	530 ± 27.5 a	507 ± 31.9 ab	463 ± 47.0 ab	495 ± 55.3 ab	480 ± 22.6 ab	458 ± 62.6 b
Urease activity (mg NH_4_^+^–N kg^−1^ dry soil d^−1^)	482 ± 42.6 bc	637 ± 37.8 a	425 ± 63.0 cd	408 ± 58.9 cd	506 ± 33.1 b	387 ± 35.2 d	531 ± 58.9 b
Acid phosphatase activity (mg *p*NP kg^−1^ dry soil d^−1^)	1886 ± 62.5 ab	1974 ± 156 a	1825 ± 195 abc	1727 ± 251 abc	1613 ± 123 c	1676 ± 144 bc	1702 ± 116 bc
Arylsulfatase activity (mg *p*NP kg^−1^ dry soil d^−1^)	124 ± 16.8 a	127 ± 7.30 a	131 ± 21.7 a	121 ± 18.9 a	110 ± 9.60 ab	116 ± 15.7 ab	94.7 ± 12.1 b
Electron transport system activity (mg O_2_ kg^−1^ soil min ^−1^)	0.22 ± 0.06 ab	0.28 ± 0.04 a	0.26 ± 0.02 a	0.22 ± 0.02 ab	0.24 ± 0.05 ab	0.26 ± 0.02 a	0.19 ± 0.02 b

^*a*^ CK, blank control; PVT, PVC presence; PCNT, PVC and multi-walled carbon nanotubes co-presences; PCDT, PVC and nanodiamonds co-presences; PDMT, PVC and DMPP co-presences; PCNMT, PVC, multi-walled carbon nanotubes, and DMPP co-presences; and PCDMT, PVC, nanodiamonds, and DMPP co-presences. Values within the same column followed by different lowercase letters (a, b, c, d) are significantly different (*P* < 0.05), as determined by one-way analysis of variance (ANOVA) followed by the Duncan multiple range test; values sharing the same letter are not significantly different.

Despite different treatment variations, the *β*-glucosidase activities remained statistically comparable (511–556 mg *p*NP kg^−1^ d^−1^). However, the PCDMT uniquely suppressed the chitinase activity by 13.6%, relative to PVT (*P* < 0.05), while the urease activity exhibited a dichotomous pattern: PVT > CK (*P* < 0.05), but the PCNT, PCDT, PDMT, PCNMT, and PCDMT treatments maintained 60.8%–83.4% of PVT levels. The acid phosphatase activity in the seven groups showed a similar trend to the chitinase and urease activities. The arylsulfatase activities in the seven groups were 124 ± 16.8, 127 ± 7.30, 131 ± 21.7, 121 ± 18.9, 110 ± 9.60, 116 ± 15.7, and 94.7 ± 12.1 mg *p*NP kg^−1^ dry soil d^−1^, respectively, and the ETSA in the seven groups were 0.22 ± 0.06, 0.28 ± 0.04, 0.26 ± 0.02, 0.22 ± 0.02, 0.24 ± 0.05, 0.26 ± 0.02, and 0.19 ± 0.02 mg O_2_ kg^−1^ soil min^−1^, respectively.

### Plant biomass, quality, and ecosystem multifunctionality

**Fig 1:**
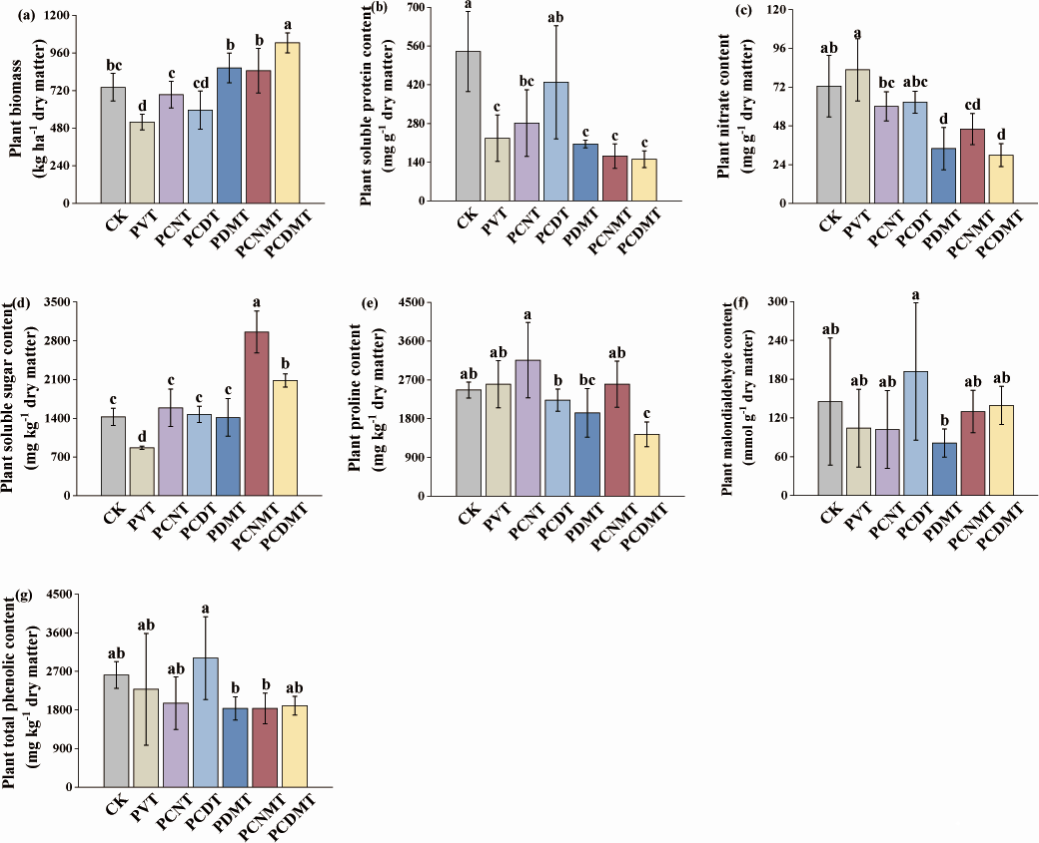
The (**a**) biomass, (**b**) soluble protein, (**c**) nitrate, (**d**) soluble sugar, (**e**) proline, (**f**) malondialdehyde, and (**g**) total phenolic contents of plants in the different treatments. CK, blank control; PVT, PVC presence; PCNT, PVC and multi-walled carbon nanotubes co-presences; PCDT, PVC and nanodiamonds co-presences; PDMT, PVC and DMPP co-presences; PCNMT, PVC, multi-walled carbon nanotubes, and DMPP co-presences; and PCDMT, PVC, nanodiamonds, and DMPP co-presences. Lowercase letters on the plots indicate significant (*P* < 0.05) differences among the seven treatments.

The PVT group induced severe biomass suppression (32.4% reduction vs CK), which was dramatically reversed by nanomaterials and DMPP interventions. The PCDMT treatment achieved maximal remediation efficacy, yielding 2.05-fold higher biomass than PVT controls (Fig. 1a).

In contrast to the CK, the PVT significantly lowered the plant soluble protein content, and the values in the PCNT and PCDT were 1.24-fold and 1.89-fold of their PVT counterpart, respectively (Fig. 1b). Relative to the CK, the PVT increased the plant nitrate content but significantly reduced the soluble sugar content, while all combined groups (PCNT, PCDT, PDMT, PCNMT, and PCDMT) significantly reversed PVT effects (Fig. 1c and d). The plant malondialdehyde and total phenolic contents displayed similar trends among the CK, PVT, PCNT, PCDT, PDMT, PCNMT, and PCDMT (Fig. 1f and g), with the largest contents in the PCDT. The PCNT, PCDT, PCNMT, and PCDMT significantly elevated the ecosystem multifunctionality by 6.0%, 6.9%, 13.2%, 8.7%, and 14.0%, in contrast to the CK (0.70 ± 0.02) (see Fig. S1 at https://doi.org/10.6084/m9.figshare.30681275).

### Soil and endophytic bacterial and fungal community diversities

**Fig 2:**
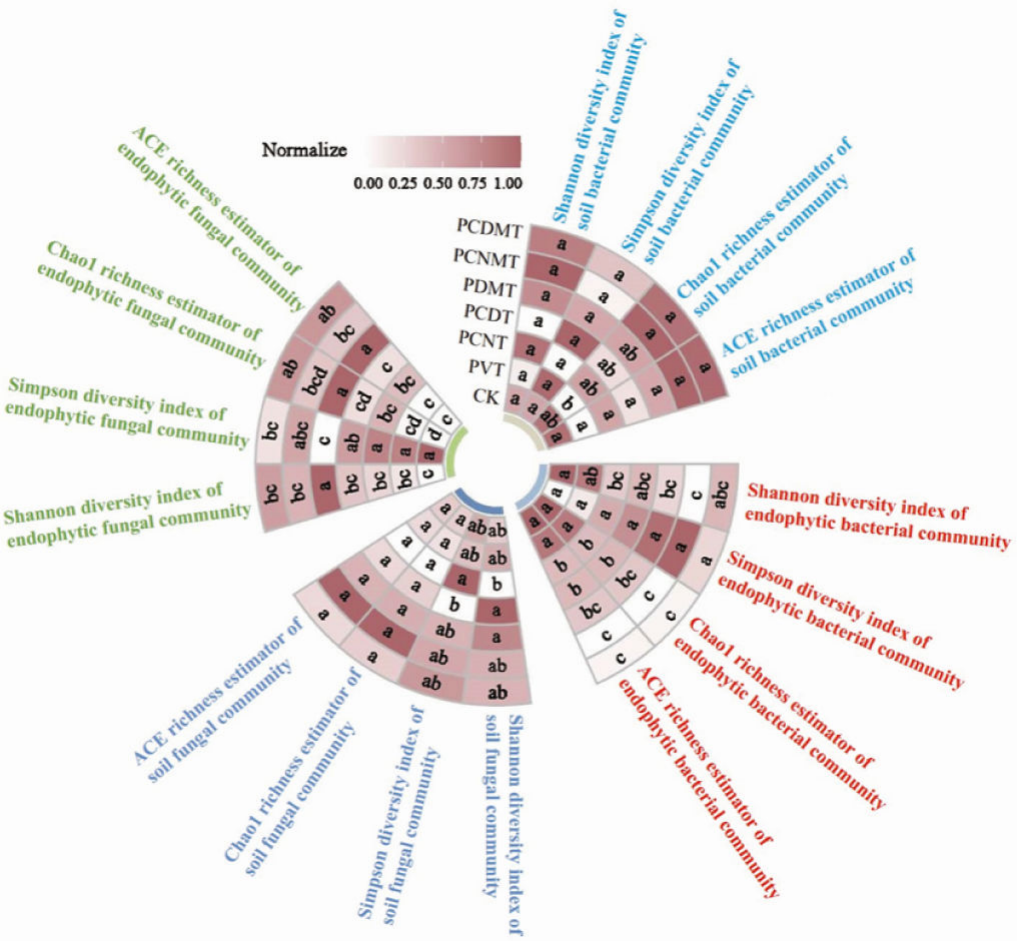
The *α*-diversities of soil and endophytic bacterial and fungal communities in the different treatments. CK, blank control; PVT, PVC presence; PCNT, PVC and multi-walled carbon nanotubes co-presences; PCDT, PVC and nanodiamonds co-presences; PDMT, PVC and DMPP co-presences; PCNMT, PVC, multi-walled carbon nanotubes, and DMPP co-presences; and PCDMT, PVC, nanodiamonds, and DMPP co-presences. The degree of shade is positively correlated with the normalized value (0–1) of each indicator. Lowercase letters on the plots indicate significant (*P* < 0.05) differences among the seven treatments.

Soil bacterial community *α*-diversity responded differentially to treatments, with the PCNMT/PCDMT increasing Chao1 indices by 18.7%–22.3% versus PVT (*P* < 0.05). Endophytic bacterial communities showed significantly lower ACE and Chao1 indices in PCNT (18.2% reduction), PCDT (19.7%), PDMT (15.4%), PCNMT (21.9%), and PCDMT (22.3%) compared to CK (*P* < 0.05). Soil fungal community *α*-diversity (ACE, Chao1, Shannon, Simpson) showed no significant differences among treatments. In contrast, endophytic fungal communities exhibited higher Chao1 richness in PVT (+12.1%), PCNT (+15.3%), PCDT (+9.8%), PDMT (+27.6%), PCNMT (+14.2%), and PCDMT (+18.4%) versus CK, with statistically significant increases in PCNT, PDMT, and PCDMT (*P* < 0.05) (Fig. 2).

### Soil and endophytic bacterial and fungal community compositions

**Fig 3:**
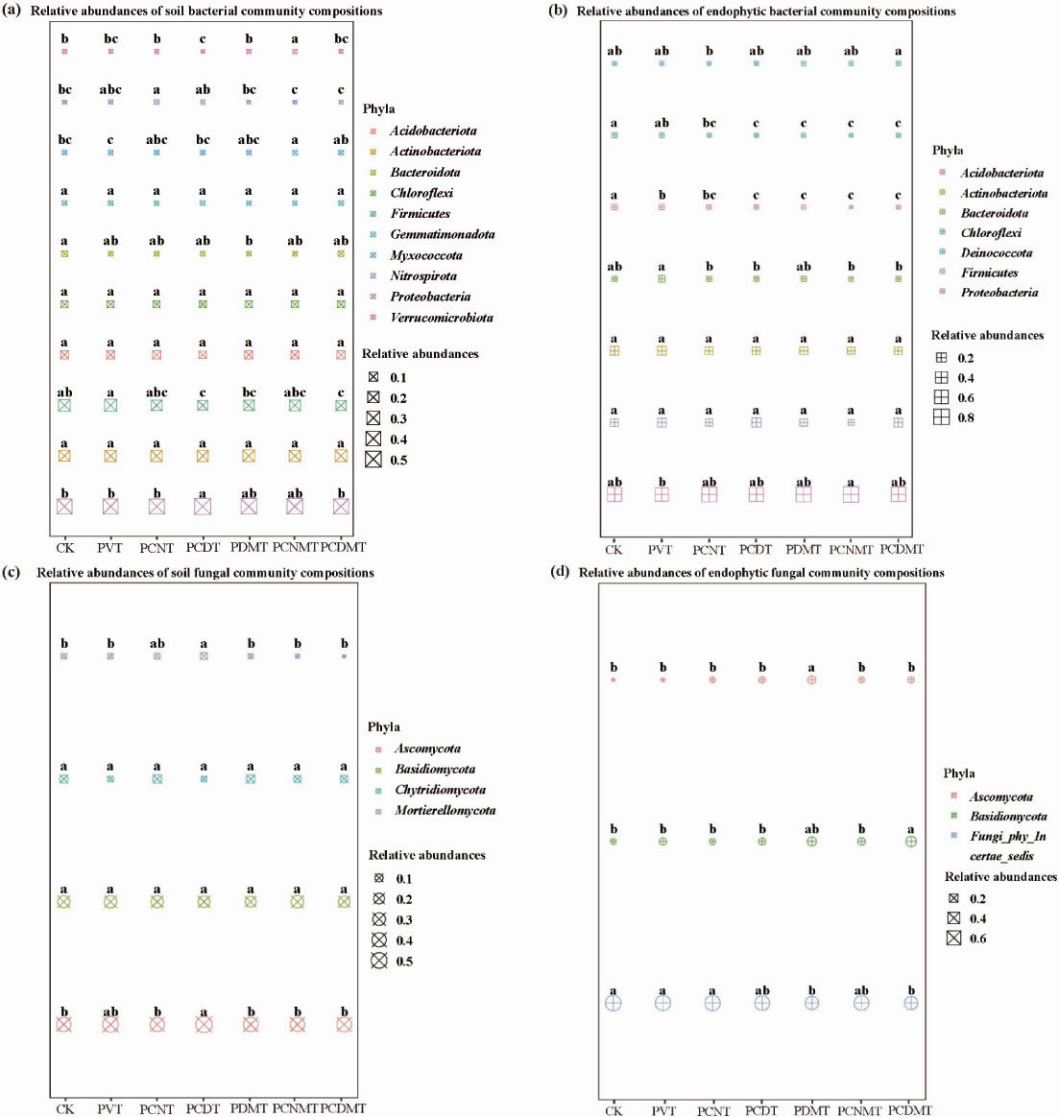
Taxonomic compositions of (**a**) soil bacterial, (**b**) endophytic bacterial, (**c**) soil fungal, and (**d**) endophytic fungal communities on the phylum level in the different treatments. CK, blank control; PVT, PVC presence; PCNT, PVC and multi-walled carbon nanotubes co-presences; PCDT, PVC and nanodiamonds co-presences; PDMT, PVC and DMPP co-presences; PCNMT, PVC, multi-walled carbon nanotubes, and DMPP co-presences; and PCDMT, PVC, nanodiamonds, and DMPP co-presences. Lowercase letters on the plots indicate significant (*P* < 0.05) differences among the seven treatments.

Relative to the CK (0.42 ± 0.03), the PCDT significantly elevated the ratio of *Proteobacteria* by 26.0%. The PVT group exhibited higher *Firmicutes* abundance than the CK counterpart, whereas the PCNT, PCDT, PDMT, PCNMT, and PCDMT reduced the abundances, with the PCDT, PDMT, and PCDMT showing the most pronounced declines. The PCNT group significantly increased the ratio of *Nitrospirota* by 55%, relative to the CK, and the PCDT and PCNMT significantly reduced and elevated the ratios of *Verrucomicrobiota* by 43% and 76%, respectively (Fig. 3a). Relative to the PVT, the PCNMT significantly elevated the ratio of *Proteobacteria*. Conversely, the ratio of *Bacteroidota* in the PVT was 2.48-fold, 3.26-fold, 3.08-fold, 2.53-fold, 5.05-fold, and 3.14-fold of those in the CK, PCNT, PCDT, PDMT, PCNMT, and PCDMT, respectively. Compared to the CK, the PVT, PCNT, PCDT, PDMT, PCNMT, and PCDMT significantly reduced the ratios of *Acidobacteriota* and *Chloroflexi*, with the PCNMT exhibiting the largest decline (Fig. 3b). The PCDT significantly elevated the ratio of *Mortierellomycota* by 58.3% versus the CK (0.05 ± 0.02). Relative to the CK, the PDMT significantly elevated the ratio of *Ascomycota* (Fig. 3d). The PDMT, PCNMT, and PCDMT significantly altered the endophytic bacterial community structures, relative to CK, and the PDMT and PCDMT significantly altered the endophytic fungal community structures (see Fig. S2; Table S7 at https://doi.org/10.6084/m9.figshare.30681275).

### Microbial community network dynamics and ecological process shifts

Relative to the CK, the PVT significantly reduced the network complexity (i.e., nodes and edges) of both soil bacterial and fungal community co-occurrence networks. In contrast, the PCNT and PCNMT treatments notably enhanced these topological parameters in bacterial community networks (*P* < 0.05), whereas the PCNT, PDMT, PCNMT, and PCDMT elevated fungal community network complexity (Table 2).(Table 2 The node and edge of the endophytic fungal community in the PDMT were significantly richer than those of the CK (Table 3).

**TABLE 2:** The topological features of co-occurrence networks of soil bacterial and fungal communities in the different treatments^*a*^

Different communities	Typological features	Treatments						
		CK	PVT	PCNT	PCDT	PDMT	PCNMT	PCDMT
Soil bacterial community	Node	412.8 ± 4.27 a	397.8 ± 4.11 b	409.8 ± 1.71 a	401.5 ± 7.05 ab	406.3 ± 6.50 ab	413.0 ± 4.32 a	405.0 ± 14.1 ab
	Edge	1099 ± 40.9 a	1016 ± 25.7 b	1075 ± 14.3 ab	1041 ± 47.9 ab	1049 ± 36.1 ab	1092 ± 32.1 a	1050 ± 73.3 ab
	Average degree	5.32 ± 0.14 a	5.11 ± 0.12 a	5.25 ± 0.05 a	5.18 ± 0.16 a	5.16 ± 0.11 a	5.29 ± 0.12 a	5.18 ± 0.20 a
	Modularity	0.75 ± 0.005 ab	0.74 ± 0.010 b	0.75 ± 0.003 ab	0.74 ± 0.004 b	0.74 ± 0.008 ab	0.75 ± 0.002 a	0.74 ± 0.010 b
Soil fungal community	Node	179.0 ± 6.68 a	165.3 ± 8.26 bc	170.3 ± 7.14 ab	156.5 ± 8.35 c	175.5 ± 4.65 ab	176.8 ± 7.37 ab	174.8 ± 8.85 ab
	Edge	310.5 ± 19.6 a	269.8 ± 24.3 bc	278.5 ± 19.9 abc	256.0 ± 18.3 c	292.3 ± 30.6 abc	305.5 ± 27.5 ab	295.0 ± 23.3 ab
	Average degree	3.47 ± 0.09 a	3.26 ± 0.14 a	3.27 ± 0.12 a	3.27 ± 0.09 a	3.33 ± 0.26 a	3.45 ± 0.23 a	3.37 ± 0.12 a
	Modularity	0.84 ± 0.006 a	0.82 ± 0.022 ab	0.84 ± 0.012 a	0.81 ± 0.014 b	0.84 ± 0.010 a	0.84 ± 0.012 a	0.83 ± 0.016 ab

^*a*^ CK, blank control; PVT, PVC presence; PCNT, PVC and multi-walled carbon nanotubes co-presences; PCDT, PVC and nanodiamonds co-presences; PDMT, PVC and DMPP co-presences; PCNMT, PVC, multi-walled carbon nanotubes, and DMPP co-presences; and PCDMT, PVC, nanodiamonds, and DMPP co-presences. Values within the same column followed by different lowercase letters (a, b, c) are significantly different (*P* < 0.05), as determined by one-way analysis of variance (ANOVA) followed by the Duncan multiple range test; values sharing the same letter are not significantly different.

**TABLE 3:** The topological features of co-occurrence networks of endophytic bacterial and fungal communities in the different treatments^*a*^

Different communities	Typological features	Treatments						
		CK	PVT	PCNT	PCDT	PDMT	PCNMT	PCDMT
Endophytic bacterial community	Node	318.0 ± 9.31 a	324.8 ± 4.99 a	292.8 ± 22.3 a	305.5 ± 14.2 a	295.8 ± 33.8 a	196.5 ± 28.7 c	235.0 ± 29.2 b
	Edge	8285 ± 47.3 a	8276 ± 21.9 a	6891 ± 1079 a	7347 ± 1024 a	6904 ± 1481 a	1764 ± 798 b	3128 ± 1003 b
	Average degree	52.1 ± 1.33 a	51.0 ± 0.81 a	46.9 ± 3.83 a	47.9 ± 4.81 a	46.3 ± 5.00 a	17.4 ± 5.52 c	26.2 ± 5.75 b
	Modularity	0.16 ± 0.006 c	0.16 ± 0.003 c	0.15 ± 0.007 c	0.15 ± 0.004 c	0.15 ± 0.009 c	0.28 ± 0.052 a	0.24 ± 0.034 b
Endophytic fungal community	Node	52.0 ± 6.98 c	50.5 ± 12.4 c	55.0 ± 12.0 bc	60.0 ± 7.66 abc	72.3 ± 5.12 a	65.4 ± 2.60 ab	67.8 ± 5.44 ab
	Edge	50.3 ± 22.8 bc	46.8 ± 30.6 c	53.5 ± 20.8 bc	67.0 ± 20.0 bc	126.0 ± 10.2 a	70.8 ± 8.62 bc	82.5 ± 17.3 b
	Average degree	1.88 ± 0.64 b	1.73 ± 0.65 b	1.88 ± 0.41 b	2.20 ± 0.45 b	3.49 ± 0.13 a	2.24 ± 0.12 b	2.42 ± 0.34 b
	Modularity	0.73 ± 0.129 ab	0.79 ± 0.092 a	0.76 ± 0.047 a	0.69 ± 0.112 ab	0.61 ± 0.087 b	0.72 ± 0.043 ab	0.70 ± 0.025 ab

^*a*^ CK, blank control; PVT, PVC presence; PCNT, PVC and multi-walled carbon nanotubes co-presences; PCDT, PVC and nanodiamonds co-presences; PDMT, PVC and DMPP co-presences; PCNMT, PVC, multi-walled carbon nanotubes, and DMPP co-presences; and PCDMT, PVC, nanodiamonds, and DMPP co-presences. Values within the same column followed by different lowercase letters (a, b, c) are significantly different (*P* < 0.05), as determined by one-way analysis of variance (ANOVA) followed by the Duncan multiple range test; values sharing the same letter are not significantly different.

The PVT tended to inhibit the heterogeneous selection process but to promote the undominated process in the soil bacterial community. Conversely, the PCNT, PCDT, PDMT, PCNMT, and PCDMT reversed these trends, enhancing heterogeneous selection while reducing undominated process (see Fig. S4 at https://doi.org/10.6084/m9.figshare.30681275).

### Plant-soil-microbe interactions

**Fig 4:**
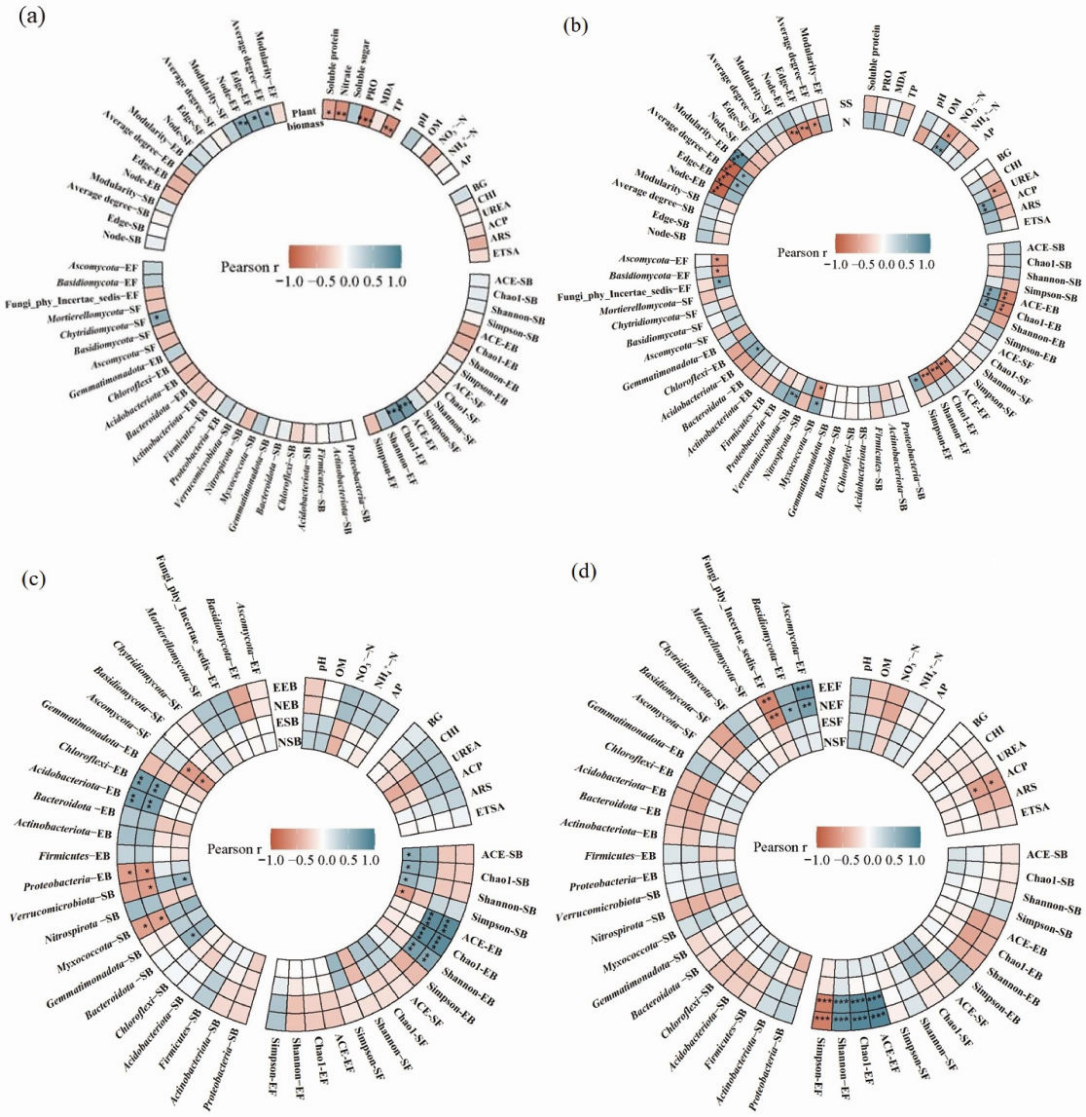
The correlation analyses reveal the relationships among the (**a**) plant biomass, (**b**) nitrate and soluble sugar contents, (**c**) node and edge of soil and endophytic bacterial community co-occurrence networks, and (**d**) node and edge of soil and endophytic fungal community co-occurrence networks, and another plant quality indicators, soil biotic and abiotic properties, *α*-diversity, composition and stabilization of soil and endophytic bacterial and fungal communities. N, nitrate; SS, soluble sugar; PRO, proline; MDA, malondialdehyde; TP, total phenolic; OM, organic matter; AP, available phosphorus; BG, *β*-glucosidase; CHI, chitinase; UREA, urease; ACP, acid phosphatase; ARS, arylsulfatase; ETSA, electron transport system activity; SB, soil bacterial community; EB, endophytic bacterial community; SF, soil fungal community; EF, endophytic fungal community; NSB, node in soil bacterial community; ESB, edge in soil bacterial community; NEB, node in endophytic bacterial community; EEB, edge in endophytic bacterial community; NSF, node in soil fungal community; ESF, edge in soil fungal community; NEF, node in endophytic fungal community; and EEF, edge in endophytic fungal community. The significant differences were accepted at *P < 0.05, **P < 0.01, ***P < 0.001.

The plant biomass exhibited significant positive correlations with soil physicochemical traits (pH, NO_3_^−^–N), plant metabolites (soluble sugar, nitrate), and the *α*-diversities (ACE and Chao1) and stabilizations (nodes and edges) of endophytic microbial communities (Fig. 4a). The plant nitrate content had significant and positive correlations with the soil NO_3_^−^–N content, and the *α*-diversity and stabilizations of endophytic bacterial community, but it had notable and negative correlations with the soil pH and the α-diversity and stabilizations of endophytic fungal community (Fig. 4b). Moreover, the plant soluble sugar content was significantly and negatively correlated with the soil nitrate, urease activity, and plant nitrate content (Fig. 4b).

Soil bacterial community co-occurrence network exhibited significant positive correlations with their own *α*-diversity but negative associations with soil NO_3_^−^–N content and urease activity (Fig. 4c). For endophytic fungi, network complexity (nodes/edges) was driven by taxonomic composition, displaying significantly and positively correlations with endophytic *Ascomycota* and *Basidiomycota*, and significantly and negatively responses to soil NO_3_^−^–N content and soil-dwelling *Basidiomycota* (Fig. 4d).

**Fig 5:**
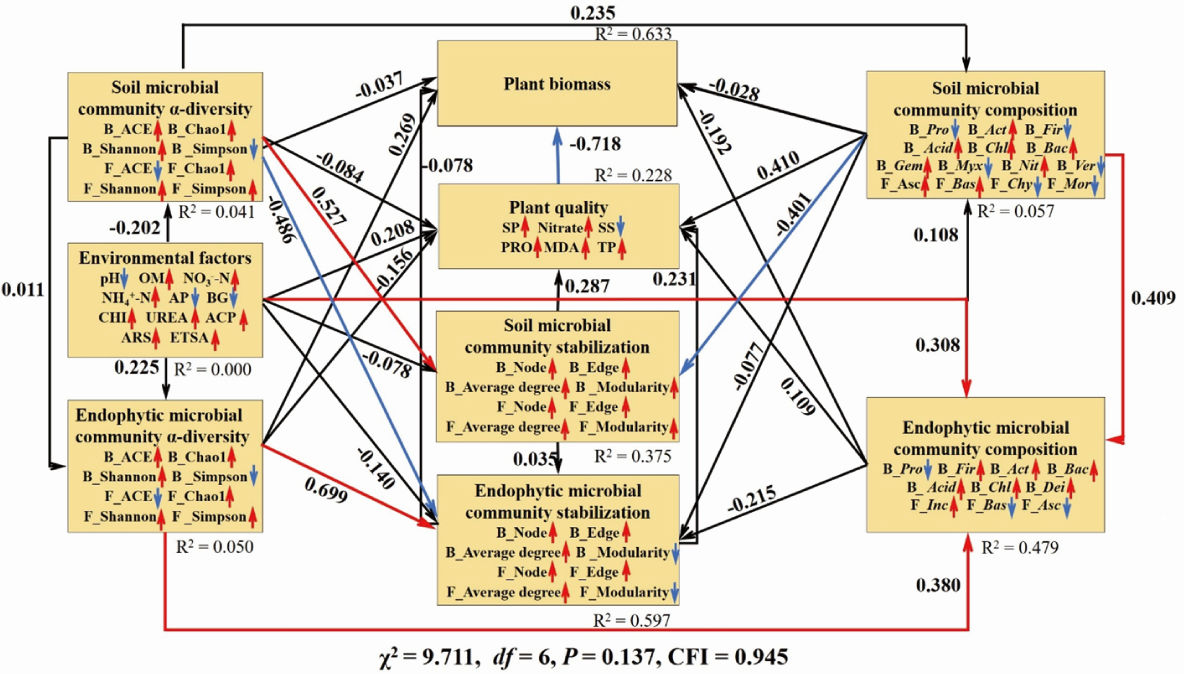
The path analysis reveals the direct or indirect linkages among plant biomass and quality, soil environmental factors, soil, and endophytic microbial communities. SP, soluble protein; B, bacterial; F, fungal; *Pro, Proteobacteria; Act, Actinobacteriota; Fir, Firmicutes; Acid, Acidobacteriota; Chl, Chloroflexi; Bac, Bacteroidota; Gem, Gemmatimonadota; Myx, Myxococcota; Nit, Nitrospirota; Ver, Verrucomicrobiota; Dei, Deinococcota; Asc, Ascomycota; Bas, Basidiomycota; Chy, Chytridiomycota; Mor, Mortierellomycota*; and *Inc*, Fungi_phy_Incertae_sedis. The red lines represented the significantly positive correlation, and the blue lines represented the significantly negative correlation. The significant differences were accepted at *P < 0.05, **P < 0.01, ***P < 0.001.

The path analysis (χ^2^ = 9.711, *df* = 6, *P* = 0.137, CFI = 0.945) demonstrated that the soil microbial community stabilization was directly altered by its own *α*-diversity (Fig. 5). In addition, the endophytic microbial community stabilization depended negatively on soil microbial community *α*-diversity but positively on its own α-diversity (Fig. 5).

## DISCUSSION

### Combined carbon nanomaterials and DMPP relieved the adverse effects of microplastics on soil and endophytic bacterial and fungal communities

PVC significantly declined the stabilization of the soil bacterial community (Table 2). This destabilization can be attributed to three mechanistic pathways: (i) PVC-derived leachates (e.g., plasticizers) might directly inhibit microbial growth or reproduction (2), thereby reducing keystone species abundance and network stabilization (i.e., nodes); (ii) microplastics-induced soil nutrient alterations could affect microbial metabolism, influencing network stabilization (8). Attributed to the formation of plastisphere and redox electron transfer processes, microplastics stimulated nitrogen metabolism-related microbial activity, particularly those associated with urease activity and NO_3_^−^–N accumulation (31, 32). In our case, the observed negative correlation between soil NO_3_^−^–N and urease activity and network complexity may stem from this N-enrichment pathway (Fig. 4c), though a direct causal relationship remains to be validated. Notably, the increased NO_3_^−^–N availability can reduce microbial respiration, thereby leading to a decline in the *α*-diversity and stabilization of the soil bacterial community (33). This aligns with our observation that N-induced microbial functional simplification could lower network connectivity, though microplastic-specific effects cannot be excluded; (iii) the ecological process and community assembly are crucial for shaping microbial co-occurrence networks (34). Here, microplastic PVC might also reduce soil bacterial community stabilization by disrupting the assembly process. Specifically, PVC exposure resulted in the dominant role of ecological drift while reducing the contribution of heterogeneous selection in the soil bacterial community assembly (see Fig. S4 at https://doi.org/10.6084/m9.figshare.30681275). This shift toward stochasticity may be attributed to microplastics modifying soil physicochemical properties—potentially by providing nutrient resources or serving as novel substrate—thereby facilitating microbial stochastic processes (8). Notably, microplastics enhance stochastic processes (such as ecological drift), weakening species-specific interactions and leading to network fragility. Consistent with our results, short-term microplastic exposure has been shown to elevate the contribution of stochastic processes to bacterial community assembly, accompanied by reduced network stabilization (8). In contrast, the extra MWCNTs tend to recover the soil bacterial community stabilization to the CK level (Table 2). Based on the previous discussion on the mechanism of microplastics reducing network stabilization, we reasonably suggest that MWCNTs could enhance the stabilization of soil bacterial community by improving soil NO_3_^−^–N content, urease activity, metabolic complementarity among bacteria, and bacterial community assembly.

The combined PVC, nanomaterials, and DMPP exerted contrasting effects on endophytic microbial communities, with significantly inhibiting the *α*-diversities and stabilizations of the endophytic bacteria while promoting those of the endophytic fungi community (Fig. 2). This phenomenon indicated the “double-edged sword” effects of nanomaterials and DMPP on the endophytic microbial communities. The opposing responses of carbon nanomaterials and nitrification inhibitors on bacterial and fungal community stem from their fundamental differences in nutrient acquisition mechanisms (35). The fungi, which rely on organic nitrogen decomposition and mycelial networks, can enhance their diversity under exogenous stress by upregulating decomposition or facilitating interspecific interactions (36). Whereas the bacteria dependent on inorganic nitrogen acquisition mechanisms are highly susceptible to exogenous interference that disrupts the diversity of functional bacterial populations (37). Moreover, nanomaterials could exert antibacterial effects by inducing oxidative stress or physically disrupting bacterial membranes (38). Furthermore, endophytic bacteria typically suppress fungal growth through multiple pathways, including antimicrobial substances secretion (39), volatile organic compound release (40, 41), siderophore-mediated nutrient competition (42), and contact-dependent killing (43). Hence, reduced bacterial community diversity potentially diminishes these suppressive impacts, possibly facilitating fungal proliferation. Collectively, the above mechanisms contribute to the observed double-edged sword effect on endophytic microbial communities. Considering the adverse impacts of nanomaterials on the endophytic bacterial community, future studies should prioritize evaluating the trade-off impacts of nanomaterials and explore the most beneficial usage measures for pollution remediation.

### Combined carbon nanomaterials and DMPP relieved the toxic impacts of microplastics on plant biomass and quality

Our study suggested that microplastics PVC exposure significantly reduced the plant biomass, while the extra MWCNTs, nanodiamonds, and DMPP significantly alleviated this toxic impact (Fig. 1). The alterations are attributed to variations in soil pH, nitrate dynamics, plant quality, and endophytic microbial communities, with soluble sugar and nitrate contents significantly correlated with plant biomass (Fig. 4a), consistent with previous studies (44). Soluble sugars, essential indicators for evaluating plant abiotic stress responses (45), influence the plant biomass by regulating metabolic pathways (46). In the present study, the PVC significantly reduced the soluble sugar content (Fig. 1d), potentially via two pathways: (i) inducing environmental stress through lowering soil pH, and (ii) disrupting sugar-metabolizing enzyme activity through releasing toxic substances (3, 47). Conversely, extra carbon nanomaterials significantly elevated the soluble sugar content by promoting nutrient uptake/transport and relieving environmental stress (48–50), while extra DMPP elevated the soluble sugar by inhibiting nitrification (19, 51). In addition, the PVC increased the plant nitrate content, which was reversed by extra MWCNTs, nanodiamonds, and DMPP (Fig. 1c). This aligns with the known link between soil acidification (via H^+^ ions influx) and stimulated plant NO_3_^−^–N uptake (52), and the role of soluble sugars in regulating the expression of nitrate transporters (53, 54). Reduced soluble sugar may have further promoted nitrate accumulation, adversely impairing plant biomass (55).

Endophytic microbes modulated plant metabolism by altering nutrient uptake (56). Specifically, endophytic bacteria synthesize growth regulators to enhance nutrient uptake while maintaining a balance with endophytic fungi, which improve root nutrient absorption through mycorrhizal networks (57). These inherent differences in nutrient acquisition mechanisms might alter microbial competitive interactions, allowing fungi to thrive by leveraging their mycelial networks to access recalcitrant resources, while bacteria maintain dominance in labile nutrient pools, thus balancing resource allocation for plant growth (19, 35, 57). Notably, our findings further revealed that combined PVC, DMPP, and MWCNTs/nanodiamonds significantly shifted this balance: it reduced the *α*-diversity and stabilizations of endophytic bacterial community while promoting those of endophytic fungal community. This microbial community restructuring was functionally relevant, as plant biomass showed significant correlations with both *α*-diversities and stabilizations of endophytic bacterial/fungal communities (Fig. 4a). Therefore, we suggest that nanomaterial substances may influence plant growth by selectively modulating microbial composition. Notably, the observed shifts in endophytic communities raise an important question regarding their origin: whether certain rhizosphere-derived microbes migrate into the plant endosphere under microplastic stress. Future studies could explore this potential translocation, which may further illuminate the dynamic crosstalk between external and internal plant microbiomes and reveal novel mechanisms of plant-microbe resilience in contaminated environments.

### Conclusion

The microplastics PVC significantly lowered the soil bacterial community stabilization, and the extra nanomaterials could recover the soil bacterial community stabilization to the blank control level by improving the soil NO_3_^−^–N content, urease activity, metabolic complementarity among bacteria, and bacterial community assembly. Moreover, the PVC significantly declined plant biomass and quality. However, the combined carbon nanomaterials, including MWCNTs and nanodiamonds, along with the DMPP, substantially alleviate the toxic impacts of microplastics on plant biomass by altering the soil pH, plant soluble sugar and nitrate contents, and *α*-diversities and stabilizations of the endophytic bacterial and fungal communities. Interestingly, the extra MWCNTs and nanodiamonds exhibited double-edged sword effects on the endophytic microbial community: while they significantly inhibited the *α*-diversities and stabilizations of the endophytic bacterial community, they promoted the *α*-diversities and stabilizations of the endophytic fungal community. Therefore, considering the adverse impacts of nanomaterials on the endophytic bacterial community, future studies should focus on the trade-off impacts of nanomaterials and explore the most beneficial usage measures for pollution remediation. This study would provide theoretical and practical foundations to mitigate the toxic effects of microplastics on the soil-plant system.

## Data Availability

The NCBI accession number for data associated with the 112 samples is PRJNA1202721.
